# Does School Health Promotion Have Additional Value for Educational Performance? A Repeated Cross-Sectional Multilevel Study

**DOI:** 10.3390/ijerph21060767

**Published:** 2024-06-13

**Authors:** Lisanne Vonk, Iris Eekhout, Tim Huijts, Mark Levels, Maria Jansen

**Affiliations:** 1 Department of Health Services Research, Care and Public Health Research Institute (CAPHRI), Maastricht University, P.O. Box 616, 6200 MD Maastricht, The Netherlands; maria.jansen@maastrichtuniversity.nl; 2 Academic Collaborative Center for Public Health Limburg, Public Health Service South Limburg, P.O. Box 33, 6400 AA Heerlen, The Netherlands; 3 Expertise Center Child Health, Netherlands Organisation for Applied Scientific Research (TNO), P.O. Box 3005, 2301 DA Leiden, The Netherlands; iris.eekhout@tno.nl; 4 Research Centre for Education and the Labour Market (ROA), School of Business and Economics, Maastricht University, P.O. Box 616, 6200 MD Maastricht, The Netherlands; t.huijts@maastrichtuniversity.nl (T.H.); m.levels@maastrichtuniversity.nl (M.L.)

**Keywords:** educational performance, school health promotion, multilevel analysis

## Abstract

Little information is available regarding the influence of the interplay between the school context and school health promotion on educational performance. Therefore, we examined whether the variation between primary and secondary schools regarding the educational performance of students could be explained by general school characteristics, school population characteristics, and school health promotion and to what extent these factors interact. We performed multilevel analyses using existing data on 7021 primary schools and 1315 secondary schools in the Netherlands from the school years 2010–2011 till 2018–2019. Our outcomes were the final test score from primary education and the average grade of standardized final exams from secondary education. School health promotion was operationalized as having obtained Healthy School (HS) certification. For the test score, 7.17% of the total variation was accounted for by differences at the school level and 4.02% for the average grade. For both outcomes, the percentage of disadvantaged students in a school explained most variation. HS certification did not explain variation, but moderated some associations. We found small to moderate differences between schools regarding educational performance. Compositional differences of school populations, especially socioeconomic status, seemed more important in explaining variation in educational performance than general school characteristics and HS certification. Some associations were moderated by HS certification, but differences remained small in most cases.

## 1. Introduction

Dutch children are among the happiest in Europe [1], and a UNICEF report showed that the Netherlands is among the best countries to be raised [2]. Despite these accomplishments, some reported trends suggest a deterioration in basic skills like reading and math among Dutch children [3]. Educational performance is determined by a complex interplay of many different factors [4,5,6,7], and numerous studies have been conducted on this topic [8,9,10,11]. Common literature findings have identified socioeconomic status (SES) as an important factor in relation to educational performance [8,12,13,14,15], but many other important factors have been identified as well, e.g., the influence of parents [4,10], migration background [16,17], teacher quality [6], general intelligence [18], the role of peers [19,20], and the school climate [7,9].

In addition to these factors, several studies suggest that a healthy diet and physical exercise patterns are also related to better educational performance of students [21,22,23,24,25]. This may be because healthy behaviors are associated with higher self-esteem [26] and better mental health [27], which are in turn related to better educational performance [28,29]. However, a considerable share of Dutch primary and secondary school students do not adhere to the minimal guidelines for fruit and vegetable consumption and physical activity [30,31,32]. As the environment and surroundings of a child play an important role in shaping these unhealthy habits [33,34,35], the World Health Organization (WHO) developed the Health Promoting Schools (HPS) framework, a whole-school approach, to create healthier school environments worldwide. Despite the fact that many health behaviors, such as dietary intake and physical activity, are also related to many factors [36], such as SES [37,38], previous research suggests that school health promotion in line with the HPS framework can be successful in promoting healthier behaviors, such as increasing fruit and vegetable intake, and (time spent on) physical activity [39,40]. But context matters: studies by Bartelink et al. [41,42] highlighted the importance of taking into account the school context when examining the impact of school health promotion, since its association with health behaviors was moderated by different factors, such as SES and organizational factors.

School health promotion programs’ primary goal is to improve children’s health behaviors, but may in the long run also contribute to their educational performance [43,44]. Even though the association between school health promotion and health behaviors has been examined in many studies, its effectiveness on educational performance is less clear, since there are few empirical studies available on the impact of programs in line with the HPS framework on educational performance [40,45]. However, some evidence is available for separate interventions, but these studies have contradicting results [46,47,48]. The results of a systematic review by Rasberry et al. [48] showed that physical education in primary or secondary school was associated with better educational performance in several studies, but not in others. Golsteyn et al. [46] even showed that a school-based intervention to stimulate physical activity in primary school students negatively affected overall educational performance. These results seem contradictory, but as we mentioned, both educational performance and health behaviors are determined by the interplay of multiple factors. Therefore, the potential role of school health promotion in improving educational performance is complicated [5,41,49]. Since educational outcomes are rarely included in evaluation studies of school health promotion [45], little information is available regarding the complex interplay between the school context and school health promotion on educational performance.

To assess the extent to which school health programs contribute to explaining educational outcomes in the Netherlands, we first explored how much variation in educational outcomes is determined by schools. We then aimed to see how much of this variation is explained by school health promotion. Therefore, in this study, we contribute to the existing literature by answering two research questions: (1) To what extent can the variation between primary and secondary schools in the Netherlands regarding educational performance be explained by differences between schools regarding general school characteristics, school population characteristics, and school health promotion? (2) To what extent is the association between general school and school population characteristics and educational performance moderated by school health promotion? To answer these questions we used a large-scale register-based data set on almost all students in Dutch education, with results on high-stakes tests as outcome variables.

## 2. Materials and Methods

### 2.1. Participants

This study had a repeated cross-sectional multilevel design. Data from the *Netherlands Cohort Study on Education* (NCO) on all publicly funded primary and secondary schools in the Netherlands, except for special education, were used. These data included educational performance of students, background information, and general school and school population characteristics [50,51]. For primary education, test scores are registered, and for secondary education, the average grade for the centralized exams is registered (including the grades of potential re-sits). Additionally, data on healthcare costs were used from Vektis [52] and data in regard to school health promotion were obtained from the HS organization. We combined all data using encrypted school and student identifiers.

We included primary and secondary school students in their final year in the school years 2010–2011 until 2018–2019. For the school year 2018–2019, we used preliminary data for various student characteristics, such as household income and educational attainment of the parents. In the Netherlands, primary schools have six grades, and afterwards, students go to secondary school, which consists of several tracks: pre-vocational secondary education (vmbo), senior general secondary education (havo), and pre-university education (vwo). Vmbo encompasses four different tracks, from practical occupation-oriented (vmbo-bb) to theoretical occupation-oriented (vmbo-tl). Vmbo takes four years to complete, havo five years, and vwo six years. We excluded special primary education and practical education *(praktijkonderwijs)*. For secondary education, data from students were registered by the NCO from 2007–2008 onwards; therefore, final year data for students who attended vmbo were included from the school year 2010–2011 onwards, for students who attended havo from the school year 2011–2012 onwards, and for students who attended vwo from the school year 2012–2013 onwards. Secondary school students (N < 10) who followed a track that was not in line with the school’s educational structure were excluded as well. We also excluded students who lived in an institutionalized household during their final year and students with no outcome data (11.7% for primary education and 3.3% for secondary education). Lastly, we excluded schools with (outcome) data from less than five students in one school year. This resulted in 1,455,153 student observations in our final analyses for primary education, and 1,595,838 student observations for secondary education.

### 2.2. Instrumentation and Procedure

***Outcomes.*** For primary education, our outcome was the test score for the end of primary school test. The ‘Cito-test’ and its successor the ‘Centralized final test’ (Centrale Eindtoets) are the most common types and test scores can range from 501 to 550. The scores of other tests, i.e., Route 8, AMN, DIA, and IEP, were harmonized to the metric of the Centralized final test using the percentile distribution of each test and its relation to a common latent variable. Note that there are a few rarely used tests that were not calibrated to the common latent variable and could not be harmonized. Scores on these tests (4.4%, not including the category ‘other tests’) were entered as missing values in the analyses. For secondary education, our outcome was the average grade of centralized and standardized exams that students from vmbo, havo, and vwo take in their final year. Students can obtain grades between 1 and 10. More elaborate information regarding our outcomes is provided in Appendix A.

***General school characteristics.*** All general school characteristics can vary per school year. For primary education, we included the following characteristics: urbanicity (high (≥1500 addresses/km^2^), medium, and low (<1000 addresses/km^2^)); school size (i.e., number of students); school type (public, independent non-denominational, Catholic, Protestant, Islamic, anthroposophic, collaboration between Catholic and Protestant, and other); and the school year (ranging from 2010 to 2018, with the year indicating the start of the school year). For secondary schools, we included the same characteristics, but for school type we distinguished public, independent non-denominational education, Catholic, Protestant, collaboration between Catholic and Protestant, collaboration religious and non-religious, and other. We also indicated whether particular tracks were offered by the school, using five separate dummy variables (vmbo-bb/kb, vmbo-gl/tl, havo, vwo, and practical education).

***School population characteristics.*** We included the following school population and student characteristics: the percentage of disadvantaged students (i.e., students with two lower-educated parents, based on the whole school population. Schools receive a subsidy from the government for students with two lower-educated parents.); age (in months on 1st May, standardized by subtracting the mean age); highest educational attainment of the mother and the father (low, medium, and high) [53]; socioeconomic category of the mother and the father (employed, receiving benefits, and inactive); and the highest known household income (low (≤25th percentile), medium, and high (≥75th percentile), based on the household incomes of the Dutch population); household composition (living with both parents/one parent/without parents); migration background (native, first generation, or second generation); and healthcare costs covered by obligatory basic health insurance (which were cut off at the 90th percentile as low and high costs). For healthcare costs, calculations differed per school year, since Dutch healthcare policy has changed over time. More elaborate information regarding the healthcare costs is included in Appendix A. For secondary education, we also included the percentage of students that lived in high-poverty areas (these students will also be referred to as disadvantaged students) and students’ educational track (vmbo-bb/kb, vmbo-gl/tl, havo, or vwo). For primary education, we also included the type of end of primary school test (Centralized final test, Cito-test, Route 8, AMN, DIA, or IEP). If a student had no information regarding the type of test and no test result, we replaced the missing value with ‘no test’.

***School health promotion.*** The current study is part of a large research project that evaluates the Dutch HS program [54]; therefore, we included several characteristics related to this program. The HS program facilitates schools in stimulating healthy habits among their students [44] by focusing on health education, physical and social school environments, identifying students who need additional attention or referral, and healthy school policy [44]. We determined whether a school was an *HS* or not and which topic certificates the school possessed. During our study period, schools could obtain a topic certificate for nutrition, physical activity, well-being, relationships and sexuality, and smoking, alcohol and drug prevention. For primary schools, there were additional topic certificates for physical safety, environment and nature, and hygiene, skin and teeth. If the school receives the topic certificate, the school automatically obtains the HS program certificate as well. A school was categorized as an HS in the school year the program certificate was obtained, and three school years afterwards. We also determined whether a school had ever been an HS, i.e., within our study period, and the number of years a school has or had been an HS for every school year separately. The degree of implementation (adherence) of the HS program was unknown, but HS certification, i.e., the HS program certificate and the topic certificates, was used as a proxy for implementing the minimum requirements. From the school year 2015–2016 onwards, we registered whether a school obtained more intensive support to implement the HS program for one of the health topics. This support was also provided in the school year 2014–2015, but data were missing in this study since no encrypted school identifiers were available. Where the location code of the school identifier was missing for HS certification or the support, and we could only identify one school location, we assumed the data concerned the same school location. More elaborate information on the HS program and related support is provided in Appendix B.

### 2.3. Data Analysis

Data were analyzed using multilevel regression analyses, using the 4.1.3 R version [55]. Missing values were imputed using multiple imputation with chained equations using the mice package [56]. We used five imputations and ten iterations. Whether a school was an HS was determined based on the separate topic certificates for every school year. Therefore, we did not include the HS indicator, whether a school had ever been an HS, or the number of years a school has or had been an HS in our imputation model. We included the estimated variance in test scores and average grade per school as auxiliary variables to incorporate the school-level variation. We also included sex of the child, the Public Health Service of the area of the school, and the teacher advice (for primary education) in the imputation model as auxiliary variables. Students in the 6th grade receive teacher advice for secondary education, e.g., havo, based on their overall educational performance. Some variables were (further) categorized after the imputation (see Appendix A).

Our model consisted of three levels: students were nested in school years and school years were nested in schools. We first examined the variation in educational performance between and within schools in a random intercept model (the null model) with the intraclass correlation coefficient (ICC) [57]. We calculated the ICC for the school level and for the school year level using two formulas:

$${ICC}_{school}=\frac{\sigma_{school}^{2}}{\sigma_{school}^{2}+\sigma_{school\;year}^{2}+\varepsilon },$$

$${ICC}_{school\;year}=\frac{\sigma_{school\;year}^{2}}{\sigma_{school}^{2}+\sigma_{school\;year}^{2}+\varepsilon },$$

In these formulas, $\sigma_{school}^{2}$ represents the estimated variance at the school level, $\sigma_{school\;year}^{2}$ the estimated variance at the school year level, and ε the residual variance. Next, we examined which characteristics explained ≥10% of the variation by adding each variable univariately to the null model [58,59]. If less than ten schools had obtained a specific topic certificate, we did not include the topic certificate separately in our analyses to examine how much of the variation was explained. The characteristics that explained ≥10% were added together in the model to determine the amount of variation between schools and school years that was explained by these variables. As a next step, we examined interactions between the characteristics that explained differences between schools and the HS program certificate in the random intercept model. For models including cross-level interactions, we included a random slope for the lower level when possible. The relevance of significant interactions, i.e., *p* < 0.01, since we had a very large sample size, was inspected based on the effect size. We also presented the association between the HS indicator and the outcomes to better interpret the interaction analyses. For all analyses with test scores, we included the type of end of primary school test to take into account differences between types of tests. In case the models produced convergence or singularity problems, we first increased the number of iterations to 20,000, and second we changed the optimizer. In case the problems were not solved, we concluded that the analysis was not possible to perform. We compared the results of our analyses based on multiple imputations to the results of the complete case analyses.

## 3. Results

**Figure A1:**
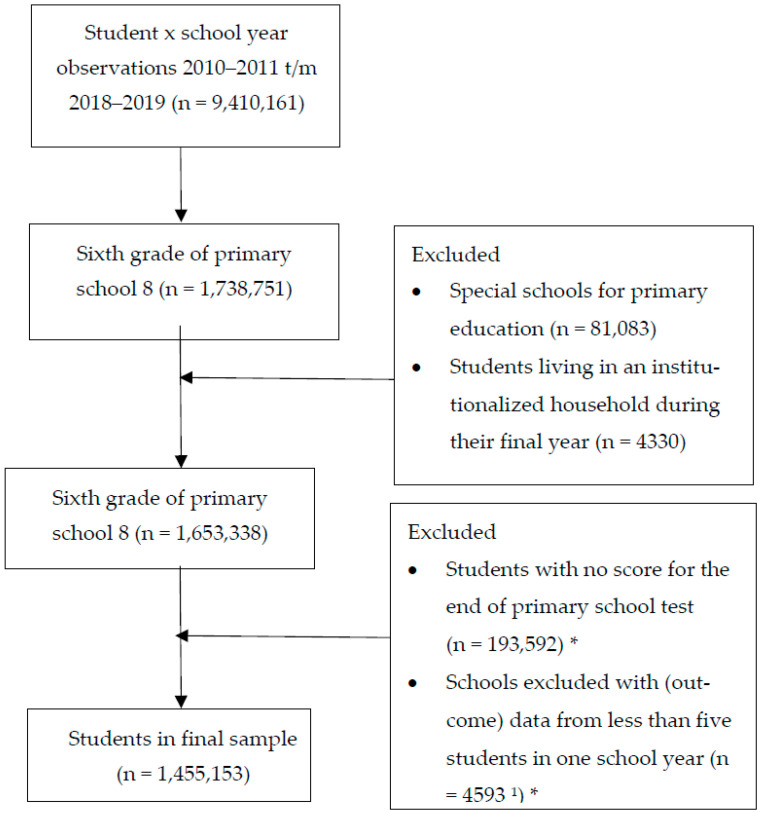
Flowchart of primary school students. * Excluded after imputation. ¹ N refers to the number of student observations.

**Figure A2:**
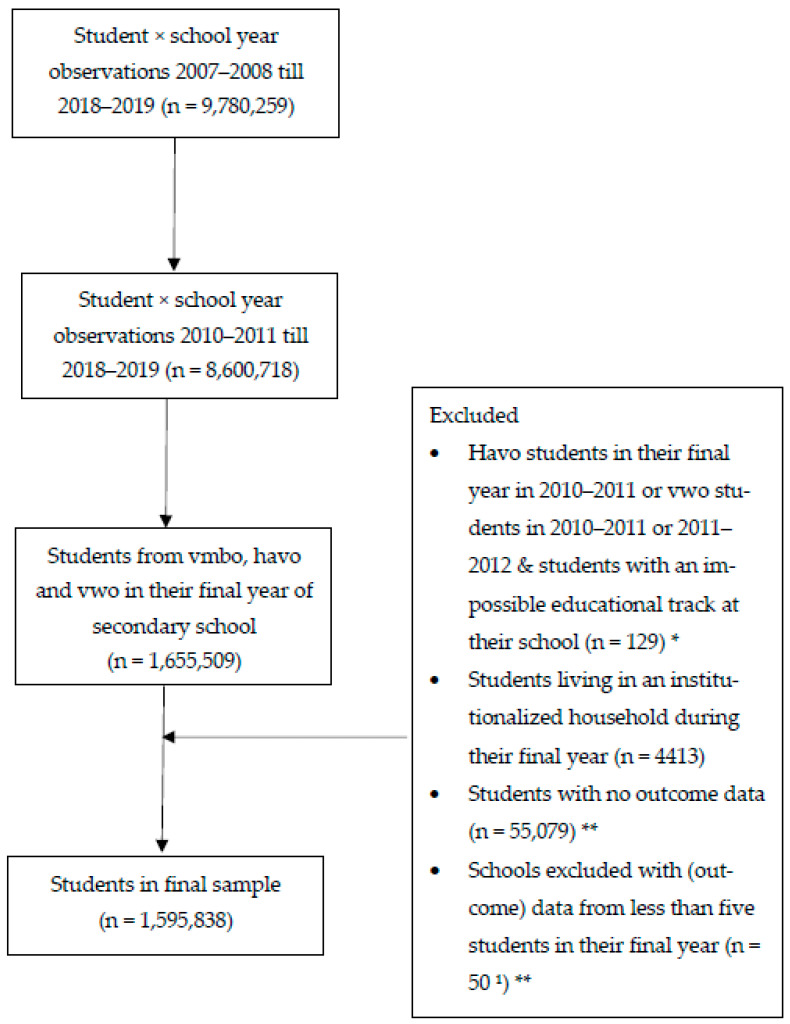
Flowchart of secondary school students. * We combined two exclusion criteria, since for one criterion, less than 10 students were excluded. We cannot report the exact number due to privacy reasons. ** Excluded after imputation. We only included outcome data of the following end of primary school tests: the Cito-test, the ‘Centralized final test’ (Centrale Eindtoets), Route 8, AMN, DIA, and IEP. ¹ N refers to the number of student observations.

Flowcharts are presented in Figure A1 and Figure A2 in Appendix C. For primary education, we included 1,653,338 student observations in our imputation model and 1,455,153 student observations in our final analyses. For secondary education, we included 1,650,967 student observations in our imputation model and 1,595,838 student observations in our final analyses. The differences are because students without outcome data and schools with data of less than five students were excluded.

### Differences in Educational Performance

***Primary school.*** Table 1 presents characteristics at the school year level separately for schools that obtained the HS program certificate during the study period (2010–2011 till 2018–2019) at least once; we refer to these schools as certified schools and non-certified schools. In total, 1262 of 7021 primary schools (18.0%) were certified schools. When we distinguish schools as certified or non-certified schools at the school year level, students in certified schools had on average significantly lower test scores (534.1 vs. 535.2). Results of multilevel analyses of the ICC are presented in Table 2. For primary school students, 7.17% of the difference in test scores was explained by differences between schools when controlling for the type of end of primary school test. The ICC decreased by ≥10% for five characteristics, i.e., percentage of disadvantaged students in the school, highest educational attainment of the mother and the father of the student, socioeconomic category of the mother of the student, and the migration background of the student, but not for any of the included characteristics related to the HS program. When adding these five variables simultaneously to the model, the ICC decreased to 2.47%. The ICC at the school year level was 4.46% but did not decrease by ≥10% for any of the characteristics. When we summarized the characteristics of the students per school year for each school, we found significant (*p* < 0.05) differences between certified and non-certified schools for all characteristics that explained variation between schools, i.e., decreased the ICC by ≥10%.

Multilevel interaction analyses are presented in Table 3. We did not find a significant association (*p* < 0.01) between HS and the test score, but our results showed some significant interaction effects between HS and the school population characteristics. There was a positive significant association between HS and the test score for schools with no disadvantaged students (B = 0.25), and the higher the percentage of disadvantaged students, the smaller the strength of this association (B = −0.02). There was also a positive significant association between HS and the test score for students with a highly-educated father (B = 0.20), this association was lower for students with a medium-educated father (B = −0.26). Students with a second generation migration background had on average lower test scores than native students (B= −1.43), but their test scores were on average higher in schools with the HS program certificate (B = 0.41). The association between HS and the test score was stronger for second-generation immigrant students compared to native students (B = 0.41). For the complete case analyses, results led to similar conclusions as the analyses based on multiple imputations, except for one notable difference: for the analyses based on multiple imputations, there was no significant interaction effect between the highest educational attainment of the mother and HS, as opposed to the complete case analyses, i.e., where the association between HS and the test score was stronger for students with a mother with low educational attainment compared to students with a mother with high educational attainment (B = 0.55). The highest educational attainment of the mother had a high number of missing values in our final sample (i.e., 40%), and having a missing value was associated with the household income, the socioeconomic category of the mother, and the migration background of the student, which had far less missing values (i.e., <2%).

***Secondary school.*** Table 1 presents characteristics at the school year level separately for certified and non-certified schools. In total, 232 of 1315 secondary schools (17.6%) were a certified school during the study period. When we distinguish schools as being a certified or non-certified school at the school year level, students in certified schools had on average slightly, but significantly higher, average grades (6.43 vs. 6.40). Results of multilevel analyses of the ICC are presented in Table 4. For secondary school students, 4.02% of the difference in the average grade was explained by differences between schools. The ICC decreased by ≥10% for three characteristics, i.e., percentage of disadvantaged students, educational attainment of the mother, and migration background of the student, but not for any of the included characteristics related to the HS program. The ICC decreased to 3.27% when adding these variables multivariately to the model. The ICC at the school year level was 3.85% but did not decrease by ≥10% by adding any of the variables. When we summarize the characteristics of the students per school year for each school, we found significant (*p* < 0.05) differences between certified and non-certified schools for all characteristics that explained variation between schools. Multilevel interaction analyses are presented in Table 5. There was a small but significant association between HS and the average grade (B = 0.04). The association between HS and the average grade was slightly stronger for students with a mother with low educational attainment compared to students with a mother with high educational attainment (B = 0.02), and for students with a second-generation migration background compared to native students (B = 0.02). For secondary education, complete case analyses led to similar conclusions as analyses based on multiple imputations.

## 4. Discussion

This study aimed to examine to what extent differences in educational performance between primary and secondary schools in the Netherlands could be explained by differences between schools regarding general school characteristics, school population characteristics, and school health promotion, and to what extent the association between these general school and school population characteristics and educational performance is moderated by school health promotion. Our results showed that 7.17% of the total variation between schools in primary school test scores, and 4.02% of the total variation of the secondary school average grades, were accounted for by differences at the school level and, respectively, 4.46% and 3.85% of the total variation was accounted for by differences within schools over time.

These differences between schools could be partly explained by compositional differences of school populations. For both primary and secondary education, the percentage of disadvantaged students explained most of the variance between schools, indicating that the percentage of disadvantaged students has an influence on test scores and the average grades of students. This is in line with previous studies that suggest that students in high-SES schools perform better than students in low-SES schools [13,15]. Findings in the literature show that the association between the average SES of the school and the educational performance of students was found to be even stronger than the association with their individual SES [15]. These results indicate that groupings of low-SES students might generate conditions in a school that are even more unfavorable for educational performance, such as more teacher shortages and less-qualified and experienced teachers [60,61,62], and vice versa for high-SES students. Besides school-level SES, indicators of individual SES, i.e., highest educational attainment of the mother, highest educational attainment of the father, and the socioeconomic category of the mother (the last two only for primary education) [13,63], also explained differences between schools. High-SES students are more likely to be raised in a more stimulating home learning environment, e.g., by having more conversational exposure [64], which could benefit their educational performance. Lastly, we found evidence that differences in educational performance between schools can be partly explained by migration background, which is also in line with previous findings in the literature [16,65]. A possible explanation is that immigrant students are more likely to speak a different language at home. This might have a negative influence on their reading abilities, and in turn on their capabilities in other school subjects [16]. Furthermore, we found that none of the characteristics substantively explained differences in educational performance within schools over time.

Variation in educational performance between schools or within schools over time was not explained by HS certification. HS certification was used as a proxy for the degree of implementation of the HS program, but this might not differentiate schools sufficiently: non-certified schools might have implemented the program as well and exposure at the individual level in terms of dose and duration might also vary to a great extent between certified schools. However, as we mentioned before, the educational performance of students can depend on many different factors and on the interplay between these factors [4,5,6,7]. Our results indicated that other factors, i.e., school population characteristics, are more important in determining educational performance than general school characteristics and HS certification. Therefore, we examined whether the association between the included SES indicators and migration background and educational performance differed between schools with and without the HS program certificate. We found evidence for subgroup differences, since there was a (more) favorable association with the HS program certificate for primary school students with a second-generation migration background compared to native students and for students in schools with a low concentration of disadvantaged students. As we mentioned, schools with a low concentration of disadvantaged students are likely to have more qualified staff and less teacher shortages [60,61,62], which might be necessary conditions for improving the educational performance in schools with the HS program certificate. A possible explanation for migration background is that both the health and the educational performance of students with a migration background are generally worse [16,66], thus leaving more room for improvement. Furthermore, there was also a very slight difference in the association between the HS program certificate and the test score between students with a medium-educated father and students with a highly-educated father. These results imply that the HS program can have very modest educational benefits in primary education for certain schools and subgroups. For secondary education, significant effect sizes were even smaller and would probably not be noticeable in practice.

### Strengths and Limitations

Since our study included existing data of the large majority of all primary and secondary schools in the Netherlands, external validity of our findings for schools and students in the Netherlands is high. This allowed us to contribute to the existing literature by capturing multifaceted variations in school contexts across the Netherlands with regard to general school and school population characteristics. This also enabled us to provide more insight into the moderating role of these characteristics.

However, since we depended on existing data, our study had some limitations. Firstly, we were restricted in the inclusion of potentially important characteristics for our study. Therefore, we were not able to include other characteristics that might affect educational performance, such as teacher quality, school climate, and a supportive home environment [4,6,7,67], or the degree of implementation of the HS program [68]. Future studies should examine if the HS program can influence the association between these school conditions and educational performance under different forms and degrees of implementation across multiple dimensions. Secondly, the use of registration data has the advantage of a large coverage of the population, however, this comes with a price. Registration data is prone to missingness, especially when data from different sources are combined. Multiple imputation is a state-of-the art method to deal with missing data and enables the optimal use of available information. To explore the effect of our missing data, we compared our results to a complete case analysis. Most of our study results were in line, except that the complete case analysis showed a significant interaction between the highest educational attainment of the mother and the HS program certificate on the test score. This finding may be due to having a selective sample, due to the high amount of missing data in the highest educational attainment (i.e., 40%) in the complete case analysis. Moreover, the missing data in educational attainment were related to information on household income, the socioeconomic category of the mother, and the migration background of the student. Including important auxiliary variables in the imputation model improves the imputation quality. For that reason, we relied on the results based on multiple imputation. Thirdly, in cases where schools merged or split, this was adjusted in the HS register; therefore, not all schools could be identified as an HS, since we used data from multiple school years. This most likely caused a non-differential misclassification of exposure [69]. Fourthly, it was not possible to include a random slope in our models for cross-level interactions. For all cross-level interactions, this caused overestimating the independence among students [70]. This should be taken into account when interpreting the results.

## 5. Conclusions

For both primary and secondary education, we found small to moderate differences between schools regarding educational performance. Our results indicated that compositional differences of school populations, especially SES, are more important in determining educational performance than general school characteristics and HS certification. However, the strength and the direction of the association between school population characteristics and educational performance was moderated by the HS program certificate, but differences between groups remained small in most cases, especially in secondary schools. To increase our understanding of the association between educational performance and general school and school population characteristics and HS certification, further research should examine whether other aspects of the school environment, such as the school climate, influence the role of the HS program in promoting healthy habits and educational performance.

## Figures and Tables

**Table 1:** Descriptive statistics of sample of primary and secondary schools, presented at the school year level.

	Test Score (N = 52,655 ^1^)		Average Grade (N = 10,017 ^1^)	
	Certified chools ^2^ (N = 10,041)	Non-Certified chools (N = 42,614)	Certified chools ^2^ (N = 1953)	Non-Certified chools (N = 8064)
Schools (N)	1262	5759	232	1083
Students (N)	288,420	1,166,733	352,587	1,243,251
Test score/average grade (Mean (SD)) ^3^	534.4 (10.2) **	535.2 (9.9)	6.41 (0.7) **	6.40 (0.7)
**General school characteristics**				
Urbanicity (%) (Mean)				
High	42.7 **	36.7	53.8 *	56.6
Medium	21.8 **	18.5	21.5 **	18.2
Low	35.4 **	44.8	24.7	25.2
School size (No. students) (Mean (SD))	237 (135) **	221 (137)	910 (536) **	775 (498)
School type (%) (Mean)				
Public	36.0 **	31.4	25.7	25.4
Independent non-denominational	4.1	4.5	15.5	15.6
Catholic	35.1 **	30.4	25.2 **	20.7
Protestants	21.7 **	31.3	18.8 **	22.6
Islamic	1.5 **	0.5	-	-
Anthroposophic	^4^	0.7	-	-
Collaboration Catholic and Protestants	1.0	0.9	10.7	9.5
Collaboration	-	-	2.3 **	4.4
Rest	^4^	0.2	1.8	1.7
*Educational structure of the school (yes) (%) (Mean)* ^5^				
Vwo	-	-	50.4 **	46.5
Havo	-	-	52.8 **	46.5
Vmbo-gl/tl	-	-	91.6 *	89.9
Vmbo-bb/kb	-	-	65.9 **	59.7
Practical education	-	-	2.6	2.4
**School population characteristics (Mean (SD))** ^6^				
Disadvantaged students (%)	14.7 (15.0) **	10.4 (11.9)	13.8 (20.4) **	17.0 (24.7)
Age (months)	146.5 (1.9) **	146.2 (1.8)	203.8 (6.6)	204.0 (7.2)
Highest educational attainment mother ^7^				
Percentage high	34.2 (23.2) **	40.0 (24.0)	28.2 (18.7)	28.4 (19.8)
Percentage medium	38.5 (17.6) **	39.2 (19.1)	43.1 (11.2) *	42.4 (12.6)
Percentage low	27.4 (23.0) **	20.8 (20.1)	28.7 (16.4)	29.2 (18.2)
Highest educational attainment father ^8^				
Percentage high	38.4 (24.1) **	43.9 (24.6)	35.3 (19.4)	35.6 (20.7)
Percentage medium	37.5 (18.5)	37.5 (20.1)	40.3 (11.3) *	39.6 (13.0)
Percentage low	24.1 (21.6) **	18.6 (19.2)	24.4 (15.1)	24.7 (17.0)
Socioeconomic category mother				
Percentage employed	73.1 (18.7) **	77.5 (15.8)	76.9 (10.4) **	75.2 (12.8)
Percentage receiving benefits	11.9 (12.5) **	8.6 (10.4)	13.6 (8.5) **	14.2 (10.0)
Percentage inactive	10.7 (9.0)	10.5 (9.4)	9.5 (4.4) **	10.6 (6.3)
Socioeconomic category father				
Percentage employed	87.1 (13.0) **	90.6 (10.8)	88.0 (7.3) **	87.2 (9.2)
Percentage receiving benefits	11.9 (12.5) **	8.6 (10.4)	11.1 (7.0) **	11.8 (8.8)
Percentage inactive	1.0 (2.5) **	0.9 (2.4)	1.0 (1.0) *	1.0 (1.3)
Household income				
Percentage high	45.2 (21.6) **	51.7 (19.9)	54.8 (15.2)	54.4 (16.9)
Percentage medium	50.4 (19.8) **	44.7 (18.5)	42.2 (13.9)	42.3 (15.2)
Percentage low	4.5 (5.8) **	3.7 (5.2)	3.0 (2.3) **	3.3 (3.0)
Household composition				
Percentage living with both parents	74.8 (14.3) **	78.0 (13.7)	70.5 (10.8)	70.3 (11.3)
Percentage living with one parent	24.6 (14.0) **	21.4 (13.4)	27.9 (9.5)	28.4 (10.6)
Percentage living without parents	0.7 (2.0)	0.7 (2.3)	1.6 (5.8)	1.4 (2.5)
Migration background				
Percentage native	71.5 (28.5) **	79.2 (23.3)	79.1 (18.5) **	76.0 (22.3)
Percentage first generation	3.7 (6.0) **	2.7 (5.2)	3.3 (3.6) **	3.7 (4.8)
Percentage second generation	24.9 (25.9) **	18.1 (21.0)	17.6 (16.0) **	20.3 (19.2)
Healthcare costs (percentage high ^9^)	10.5 (7.6) **	9.8 (7.5)	10.5 (4.0) **	10.1 (3.8)
Educational track				
Percentage vwo	-	-	11.5 (18.7) **	13.3 (23.6)
Percentage havo	-	-	21.3 (26.9) **	19.5 (26.8)
Percentage vmbo-gl/tl	-	-	34.6 (31.6) **	36.8 (35.7)
Percentage vmbo-bb/kb	-	-	32.6 (36.5) *	30.4 (38.5)
**Characteristics related to the Healthy School program**				
*Healthy School topic certificates yes (%) (Mean)*				
Nutrition	13.0	-	13.9	-
Physical activity	19.1	-	21.8	-
Well-being	14.5	-	6.1	-
Smoking, alcohol, and drug prevention	0.7	-	4.7	-
Relationships and sexuality	2.4	-	2.5	-
Physical safety	0.6	-	-	-
Environment and nature	0.8	-	-	-
No. of years Healthy School (Mean (SD))	1.0 (1.5)	-	0.9 (1.3)	-
Support ^10^ (yes) (%) (Mean)	11.4 **	1.1	12.9 **	1.8
*No. of schools with the Healthy School program certificate*				
2010–2011	0	-	0	-
2011–2012	15	-	0	-
2012–2013	39	-	0	-
2013–2014	101	-	0	-
2014–2015	248	-	42	-
2015–2016	482	-	95	-
2016–2017	814	-	160	-
2017–2018	983	-	201	-
2018–2019	1126	-	211	-

Note: - = not applicable; * = significant differences between certified and non-certified schools (*p* < 0.05); ** = significant differences between certified and non-certified schools (*p* < 0.01). ^1^ The number of school x school year combinations with data unless otherwise stated. Data are summarized per school, for every school year separately. ^2^ Certified schools are schools that obtained the Healthy School program certificate at least once in the study period (2010–2011 till 2018–2019). ^3^ Outcomes are presented on the individual level, not the school year level. For the average grade, data were missing from < 10 students, but imputed data were included in the analyses since it was known whether they received a diploma or not. ^4^ Percentages (as well as * to indicate significant differences) are not presented due to privacy reasons. ^5^ Schools can offer multiple educational tracks. ^6^ Except for the percentage of disadvantaged students, school population characteristics are measured on the individual level, but descriptive statistics are reported at the school year level. ^7^ For primary education, the number of school x school year combinations was 10,033 for certified schools and 42,519 for non-certified schools. ^8^ For primary education, the number of school x school year combinations was 10,016 for certified schools and 42,478 for non-certified schools. For secondary education, the number of school x school year combinations was 1952 for certified schools and 8063 for non-certified schools. ^9^ The cut-off points for the final analyses are determined based on imputed data. ^10^ For primary education, the number of school x school year combinations was 8944 for certified schools and 38,044 for non-certified schools. For secondary education, the number of school x school year combinations was 1731 for certified schools and 7133 for non-certified schools. N(o) = number; SD = standard deviation.

**Table 2:** Multilevel intraclass correlations in primary schools for the test score (N = 52,655 ^1^).

	ICC School Level (%)	ICC School Year Level (%)
0 model	7.17	4.46
**General school characteristics**		
Urbanicity	6.96	4.47
School size	6.93	4.48
School type	6.81	4.48
**School population characteristics**		
% disadvantaged students	3.57 *	4.68
Age ^2^	6.67	4.97
Highest educational attainment mother ^2^	3.71 *	4.88
Highest educational attainment father ^2^	4.07 *	4.81
Socioeconomic category of the mother ^2^	6.15 *	4.52
Migration background ^2^	6.32 *	4.51
Healthcare costs ^2^	7.12	4.48
**Characteristics related to the Healthy School program**		
Healthy School	7.18	4.46
Healthy School ever	7.09	4.46
Number of years Healthy School	7.19	4.45
*Healthy School topic certificates*		
Physical activity	7.17	4.46
Well-being	7.17	4.46
Smoking, alcohol, and drug prevention	7.17	4.46
Relationships and sexuality	7.17	4.46
Environment and nature	7.17	4.46
Physical safety	7.17	4.46
All significant variables multivariately ^3^	2.47 *	-

Note: * ICC decreased by ≥ 10% after including the variable(s) in the 0 model. ^1^ Number of school x school year combinations included in the analyses. ^2^ Variables were measured at the individual level. ^3^ Only those variables were added multivariately for which the ICC decreased by ≥10% after including the variable. For the school year level, none of the characteristics explained the ICC with ≥10%. N: student observations = 1,455,153; schools = 7021. Due to convergence and/or singularity warnings, analyses with the nutrition certificate, support, household composition, household income and socioeconomic category of the father were not possible. Results are controlled for type of end of primary school test. Parameters are not shown for this control variable.

**Table 3:** Possible moderators of the HS program certificate on the test score.

	Primary Schools (N = 52,655) ^1^ Test Score	

	B	99% CI
*Model 1: Healthy School* ^2^		
Intercept	534.68	(534.57, 534.78) *
HS	0.10	(−0.07, 0.26)
*Model 2: Disadvantaged students*		
Intercept	536.10	(536.00, 536.19) *
% of disadvantaged students	−0.13	(−0.14, −0.13) *
HS	0.25	(0.04, 0.47) *
HS × % of disadvantaged students	−0.02	(−0.03, −0.01) *
*Model 3: Highest educational attainment mother* ^2^		
Intercept (high = ref)	537.93	(537.84, 538.02) *
Medium	−4.52	(−4.63, −4.41) *
Low	−7.39	(−7.47, −7.30) *
HS	0.11	(−0.11, 0.32)
HS × medium	−0.14	(−0.41, 0.13)
HS × low	0.20	(−0.03, 0.43)
*Model 4: Highest educational attainment father* ^2^		
Intercept (high = ref)	537.53	(537.42, 537.65) *
Medium	−4.18	(−4.40, −3.96) *
Low	−6.74	(−6.88, −6.59) *
HS	0.20	(0.00, 0.39) *
HS × medium	−0.26	(−0.46, −0.06) *
HS × low	0.01	(−0.21, 0.24)
*Model 5: Socioeconomic category mother* ^2^		
Intercept (employed = ref)	535.19	(535.09, 535.28) *
Receiving benefits	−3.16	(−3.23, −3.09) *
Inactive	−0.65	(−0.73, −0.58) *
HS	0.10	(−0.07, 0.27)
HS × receiving benefits	−0.09	(−0.31, 0.14)
HS × inactive	0.16	(−0.11, 0.42)
*Model 6: Migration background* ^2^		
Intercept (native = ref)	535.03	(534.93, 535.13) *
First generation	−2.14	(−2.28, −2.00) *
Second generation	−1.43	(−1.49, −1.37) *
HS	0.00	(−0.17, 0.17)
HS × first generation	0.28	(−0.13, 0.70)
HS × second generation	0.41	(0.21, 0.62) *

Note: ^1^ School x school year combinations. ^2^ The association between HS and the test score. * = significant (*p* < 0.01); CI = confidence interval; HS = the Healthy School program certificate. N: students = 1,455,153; primary schools = 7021. Results are controlled for type of end of primary school test (Centralized final test = ref). Parameters are not shown for this control variable. For the cross-level interactions, it was not possible to add a random slope for the lowest level due to convergence and/or singularity problems.

**Table 4:** Multilevel intraclass correlations in secondary schools for the average grade (N = 10,017 ^1^).

	ICC School Level (%)	ICC School Year Level (%)
0 model	4.02	3.85
**General school characteristics**		
Urbanicity	3.99	3.85
School size	4.04	3.84
School type ^2^	3.97	3.85
*School structure*		
Vwo	3.99	3.84
Vmbo-gl/tl	3.70	3.88
Vmbo-bb/kb	4.00	3.85
Practical education	4.02	3.85
**School population characteristics**		
% disadvantaged students	3.49 *	3.87
Age ^3^	4.51	3.98
Highest educational attainment mother ^3^	3.56 *	3.73
Highest educational attainment father ^3^	3.64	3.76
Socioeconomic category of the mother ^3^	3.89	3.85
Socioeconomic category of the father ^3^	3.89	3.85
Household income ^3^	3.84	3.84
Household composition ^3^	4.00	3.84
Migration background ^3^	3.54 *	3.88
Healthcare costs ^3^	4.02	3.85
Educational track student ^3^	3.78	3.78
**Characteristics related to the HS program**		
HS	4.02	3.83
HS ever	4.02	3.85
Number of years HS	4.02	3.84
*HS topic certificates*		
Nutrition	4.01	3.84
Physical activity	4.03	3.83
Well-being	4.02	3.84
Smoking, alcohol, and drug prevention	4.02	3.85
Relationships and sexuality	4.02	3.84
Support	4.02	3.84
All significant variables multivariately ⁴	3.27 *	-

Note: * ICC decreased by ≥10% after including the variable(s) in the 0 model. ^1^ Number of school year x school combinations included in the analyses. ^2^ Analysis was performed with a different optimizer. ^3^ Variables were measured at the individual level. ⁴ Only those variables were added multivariately for which the ICC decreased by ≥10% after including the variable. For the school year level, none of the characteristics explained the ICC with ≥10%. N: student observations = 1,595,838; schools = 1315. Due to convergence and/or singularity problems, the analysis with havo was not possible. HS = the Healthy School program certificate.

**Table 5:** Possible moderators of the HS program certificate on the average grade.

	Secondary Schools (N = 10,017) ^1^ Average Grade	
	B	99% CI
*Model 1: Healthy School* ^2^		
Intercept	6.39	(6.38, 6.41) *
HS	0.04	(0.02, 0.06) *
*Model 2: Disadvantaged students*		
Intercept	6.43	(6.41, 6.44) *
% of disadvantaged students	0.00	(0.00, 0.00) *
HS	0.03	(0.01, 0.05) *
HS × % of disadvantaged students	0.00	(0.00, 0.00)
*Model 3: Highest educational attainment mother* ^2^		
Intercept (high = ref)	6.50	(6.49, 6.52) *
Medium	−0.12	(-0.13, −0.11) *
Low	−0.20	(−0.21, −0.20) *
HS	0.02	(0.00, 0.04)
HS × medium	0.01	(−0.01, 0.02)
HS × low	0.02	(0.01, 0.04) *
*Model 4: Migration background* ^2^		
Intercept (native = ref)	6.44	(6.43, 6.45) *
First generation	−0.19	(−0.20, −0.18) *
Second generation	−0.17	(−0.18, −0.17) *
HS	0.03	(0.01, 0.05) *
HS × first generation	0.03	(0.00, 0.06)
HS × second generation	0.02	(0.01, 0.03) *

Note: ^1^ School x school year combinations. ^2^ The association between HS and the average grade. * = significant (*p* < 0.01); CI = confidence interval; HS = the Healthy School program certificate. N: students = 1,595,838; secondary schools = 1315. For the cross-level interactions, it was not possible to add a random slope for the lowest level due to convergence and/or singularity problems.

## Data Availability

Restrictions apply to the availability of these data. Data were obtained from Statistics Netherlands (CBS) and Healthy School and are available at microdata@cbs.nl and info@gezondeschool.nl, with the permission of CBS and Healthy School.

## Appendix A

**Outcome for primary education**

Our outcome for primary education was the test score for the end of primary school test. As of the school year 2014–2015, primary schools are obligated by law to implement an end of primary school test in 6th grade [71]. Nevertheless, the majority of schools already used this test before this time. Several approved primary school tests are available, and each primary school test has its own test methodology and results in its own score on its own metric. The ‘Cito-test’ and its successor the ‘Centralized final test’ (Centrale Eindtoets) are the most common, and both tests are scored on the same metric that ranges from 501 to 550. The test scores of other tests, i.e., Route 8, AMN, DIA, and IEP, were harmonized to the metric of the Centralized final test (and the Cito-test) using the percentile distribution of each test and its relation to a common latent variable [72]. The common latent variable is based on an anchor of 60 questions that are part of each test since 2018 to enable comparability between and within tests over time [73]. For our study, we used the data of the school year 2018–2019. Note that there are a few rarely used tests that do not use the anchor questions and could not be harmonized. Students with scores on these tests (4.4%, not including the category ‘other tests’) were entered as missing values in the analyses.

**Healthcare costs**

To calculate the total healthcare costs that are covered by obligatory basic health insurance, we used data on healthcare costs from Vektis [52]. We included the following categories: general practitioner (without the registration fee), pharmacy, hospital care, dental care, international health care costs, paramedical care, birth care, multidisciplinary care, and ‘other’. From 2015 onwards, we also included costs for district nursing and sensory care. For 2018, we included costs for short-term stays in healthcare facilities (other than hospitals). These costs were in 2017 included in the category ‘other’. Health care data of students that were insured through a proxy holder were included as missing values due to unreliability. Calculations differed per school year since Dutch healthcare policy has changed over time. Healthcare costs for 2010 were used for the 2010–2011 school year, 2011 for 2011–2012, and so forth.

**Imputation model**

Data were analyzed using multilevel regression analyses, using the version 4.1.3 R. Missing values were imputed using multiple imputation with chained equations using the mice package [56]. We used five imputations and ten iterations. Whether a school was an HS was determined based on the separate topic certificates for every school year. Therefore, we did not include the HS indicator, whether a school had ever been an HS, and the number of years a school has or had been an HS in our imputation model. We included the estimated variance in test scores and average grade per school as auxiliary variables to incorporate the school-level variation. We also included the sex of the child, the Public Health Service of the area of the school, and the teacher advice in the imputation model as auxiliary variables. During the final year of primary school, students receive teacher advice for secondary school based on their overall educational performance. Before the 2014–2015 school year, teachers gave their advice after the end of primary school test; as of 2014–2015, teachers give it beforehand. Therefore, teachers are allowed to revise their advice to the advantage of students since 2014–2015. We used the revised teacher advice when applicable. When a student received double advice, such as havo/vwo, we categorized it as vwo, since students are allowed to enter the highest track of their advice. Indistinct advice including more than two tracks was included as missing values, since this advice was too indefinite to provide accurate information about the educational performance of students in the imputation model. The school year was included as a numeric variable in the imputation model.

**After imputation**

Some variables were (further) categorized after imputation. This was the case for household income, healthcare costs, and educational track of the student. Healthcare costs and household income (in percentiles) were included continuously in the imputation model. After imputation, household income was categorized as low (<25th percentile), medium, and high (>75th percentile), and healthcare costs were cut off at the 90th percentile as low and high costs. For the educational track, vmbo-bb and vmbo-kb were combined after imputation, as well as vmbo-gl and vmbo-tl. Age was standardized by subtracting the mean age in months. After imputation, we also excluded students with no outcome data and we excluded the schools with (outcome) data from less than five students in one school year.

## Appendix B

**The Dutch Healthy School program**

To examine the impact of school health promotion on educational performance, we included several characteristics related to the Dutch Healthy School (HS) program. The current study is part of a large research project that evaluates the Dutch HS program [54], a whole-school approach, which focusses on school context and an association with implementation and student outcomes. This program facilitates schools in stimulating healthy habits among their students, hopefully also in the long run resulting in better health and better educational performance among primary, secondary, and secondary vocational school students [44]. The HS program provides different types of support, and schools can obtain an HS program certificate if they fulfill the minimum requirements. These requirements relate to four pillars: health education, physical and social school environments, identifying students or students who need additional attention or referral, and healthy school policy [44]. These minimum requirements are specified per theme. For both primary and secondary education, we determined whether a school was an HS or not and which topic certificates the school possessed for every school year. During our study period, schools could obtain a topic certificate for the following topics: nutrition, physical activity, well-being, relationships and sexuality, and smoking, alcohol and drug prevention. For primary schools, there were additional topic certificates for physical safety, environment and nature, and hygiene, skin, and teeth. A school was categorized as an HS in the school year the certificate was obtained, and three school years afterwards. To determine the school years, we used August first as a cut-off point. We also determined whether a school had ever been an HS, i.e., during our study period, and the number of years a school has or had been an HS, for every school year separately. The degree of implementation of the HS program was unknown and can vary between schools, but HS certification was used as a proxy for implementation adherence of the minimum requirements regarding the four pillars. A school can voluntarily fill out a questionnaire for a topic certificate if they meet the requirements for all four pillars of one of the HS program’s specific health topics [44]. This self-reported questionnaire is inspected by the HS program organization and thematic specialists to determine whether the school fulfills all the criteria. If the school receives the topic certificate, the school automatically obtains the HS program certificate as well. From the 2015–2016 school year onwards, we registered whether a school obtained more intensive support to implement the HS program for one of the health topics. This includes financial aid, support from an HS adviser, and different training regarding (the implementation of) the HS program or one of the health topics (since 2017–2018). This support was also provided in the 2014–2015 school year, but data were missing in this study since no encrypted school identifiers were available.
